# Dexmedetomidine in Children on Extracorporeal Membrane Oxygenation: Pharmacokinetic Data Exploration Using Previously Published Models

**DOI:** 10.3389/fped.2022.924829

**Published:** 2022-06-27

**Authors:** Céline Thibault, Athena F. Zuppa

**Affiliations:** Department of Anesthesiology and Critical Care Medicine, Children's Hospital of Philadelphia, Philadelphia, PA, United States

**Keywords:** extracorporeal membrane oxygenation, neonates, sedation, dexmedetomidine, pediatrics, pharmacokinetics, pharmacology, analgesia

## Abstract

**Background:**

Dexmedetomidine is a sedative and analgesic increasingly used in children supported with extracorporeal membrane oxygenation (ECMO). No data is available to describe the pharmacokinetics (PK) of dexmedetomidine in this population.

**Methods:**

We performed a single-center prospective PK study. Children <18 years old, supported with ECMO, and on a dexmedetomidine infusion as part of their management were prospectively included. PK samples were collected. Dexmedetomidine dosing remained at the discretion of the clinical team. Six population PK models built in pediatrics were selected. Observed concentrations were compared with population predicted concentrations using the PK models.

**Results:**

Eight children contributed 30 PK samples. None of the PK models evaluated predicted the concentrations with acceptable precision and bias. Four of the six evaluated models overpredicted the concentrations. The addition of a correction factor on clearance improved models' fit. Two of the evaluated models were not applicable to our whole population age range because of their structure.

**Conclusion:**

Most of the evaluated PK models overpredicted the concentrations, potentially indicating increased clearance on ECMO. Population PK models applicable to a broad spectrum of ages and pathologies are more practical in pediatric critical care settings but challenging to develop.

## Introduction

Dexmedetomidine is an α2-agonist with analgesic and sedative properties. It has become a commonly used sedative in pediatric critical care settings (1). Contrary to other sedatives, it does not affect the respiratory drive and, therefore, is particularly appealing in children supported by non-invasive ventilation or to facilitate extubation (2, 3). It has also been associated with reduced delirium and withdrawal (4). Its favorable efficacy and safety profile led to the spreading of its use to other populations, including critically ill intubated children with organ dysfunctions. Dexmedetomidine is extensively metabolized *via* glucuronidation and CYP2A6 hydroxylation and inactive metabolites are excreted by the kidneys (5). Critical illness may impact dexmedetomidine pharmacokinetics (PK) notably through reduced hepatic metabolism and increased volume of distribution (V) caused by the presence of organ dysfunctions and inflammation, which may be further influenced by the presence of extracorporeal support (6–9).

Extracorporeal membrane oxygenation (ECMO) is a heart and lung support machine. It is a life-saving modality used in severely ill children with cardiac or respiratory compromise. In addition to the impact of critical illness, ECMO may influence medications' PK in numerous ways, including the need for additional fluids and blood products administration for circuit priming and direct adsorption by the circuit and the oxygenator (9). Although dexmedetomidine is not typically considered a first-line sedative in this population, it is among the most commonly used medications in children supported with ECMO (10). However, no data is available to describe its PK and guide dosing in this population. We, therefore, conducted this study, aiming to characterize the PK of dexmedetomidine in critically ill children on ECMO.

## Method

This was a prospective single-center PK study at Children's Hospital of Philadelphia. This study was approved by the Children's Hospital of Philadelphia Review Board (IRB 18-014980). Children <18 years old, treated with ECMO, and receiving a dexmedetomidine intravenous infusion per standard of care were eligible. Exclusion criteria included significant bleeding requiring massive blood products transfusions and administration of dexmedetomidine *via* other modes of administration, such as intranasal dexmedetomidine. In addition to demographics and dexmedetomidine-related data (date and time of initiation and dosing throughout sampling period as well as plasma concentrations), ECMO-related data (ECMO type, date and time of cannulation and weaning), and laboratory values of tests obtained by the treating team as part of clinical management [complete blood count, blood urea nitrogen, serum creatinine, alanine aminotransferase, aspartate aminotransferase, and international normalized ratio (INR)] were collected.

### Sample Collection and Determination of Dexmedetomidine Concentrations

Per our initial protocol, a maximum of 16 PK samples per subject (0.5 mL/sample) could be drawn at any time while on ECMO and receiving a dexmedetomidine infusion. Samples were collected at the same time as scheduled laboratory assessments. Once collected, samples were transferred into labeled heparinized tubes, and placed on ice for a maximum of 30 min before centrifugation (3,400 RPM for 15 min at 4°Celsius). Plasma was then frozen at −70° Celsius until assayed. Dexmedetomidine concentrations were determined using a previously described and validated ultra–high–performance liquid chromatography-tandem mass spectrometry assay (11). The lower limit of quantification (LLOQ) was 5 pg/mL. The inter-day precision [coefficient of variation (%)] and accuracy of the quality control samples (5, 15, 30, 300, and 1,200 pg/mL) ranged from 2.13 to 8.45% and 97 to 104%, respectively (11).

### Analysis

Our initial analysis plan included population PK model building. However, our low recruitment rate and limited number of samples precluded the development of a robust model. We therefore decided to determine whether models that were published in the literature in children who were not treated with ECMO were able to predict the data that we derived from our study. To this end, we identified six population PK models of intravenous dexmedetomidine (Table 1) (12–17) and used external validation methods to compare our observed concentrations in children on ECMO with predicted concentrations using each model and the dosing information for each subject. The models were selected because they were developed in a population including infants <1 year old, which represent most of our population, and were described in sufficient detail in the original manuscript to be reproducible. First, the parameter estimates and covariate effects described in each of the six published models were used to calculate population predicted concentrations using our cohort's individual characteristics and dosing history. This was done by fixing the PK parameters and estimated covariate effects and running the models with no additional fitting on our dataset. In the event that a covariate was not applicable to our whole population (e.g., cardiopulmonary bypass time), this covariate was only applied when relevant and omitted otherwise. This resulted in six individual datasets of population predicted concentrations.

**Table 1:** Description of the population PK models evaluated.

**Model ID**	**Population studied**	**CMT**	**Model**
1^a^ Potts et al. (12)	95 patients aged 1 week to 14 years	2	$CL=42.1\;*\;{\left(WT/70\right)}^{075}\;*\;\left(\frac{PMA^{Hill}}{{TM_{50}}^{Hill}\;+\;PMA^{Hill}}\right)\;*\;F_{\inf}$ *V*_1_ = 56.3 * (*WT*/70) *Q* = 78.3 * (*WT*/70)^0.75^ *V*_2_ = 69 * (*WT*/70)
2^b^ van Dijkman et al. (13)	6 neonates aged 0 to 23 days for model building and 11 neonates for model validation	2	$CL=42.1\;*\;{\left(WT/70\right)}^{075}\;*\;\left(\frac{PMA^{Hill}}{{TM_{50}}^{Hill}\;+\;PMA^{Hill}}\right)$ *V*_1_ = 80.4 * (*WT*/70) *Q* = 12.5 * (*WT*/70)^0.75^ *V*_2_ = 150 * (*WT*/70)
3 Greenberg et al. (14)	20 infants aged 4 days to 6.8 months	1	$CL=48.2\;*\;{\left(WT/70\right)}^{0.75}\;*\;{\left(\frac{PMA}{43.6}\right)}^{1.94}$ *V* = 106 * (*WT*/70)
4^c^ Su et al. (15)	59 infants following cardiac surgery aged 0.1 to 20 months	2	$\begin{array}{l} CL=39.4\;*\;(WT/70)0.75\;*\;\left(\frac{Age}{0.032\;+\;Age}\right)\;*\;{\left(\frac{CPB}{60}\right)}^{-0.31}\;*\;F_{shunt}\\ V_{1}=\;88\;*\;(\frac{WT}{70})\\ Q=407\;*\;(WT/70)0.75\\ V_{2}=112\;*\;(\frac{WT}{70)} \end{array}$
5 Damian et al. (16)	20 patients following liver transplant aged 1 month to 18 years	2	$CL=52\;*\;e^{-0.484\;*\;\exp\left(\eta_{cov}\right)\;*\;\left(INR-INR_{median}\right)}$ $V_{1}=\;186\;*\;\left(\frac{WT}{70}\right)$ *Q* = 246 *V*_2_ = 203 * (*WT*/70)
6^d^ James et al. (17)	354 patients following cardiac surgery aged 0 to 22 years	2	$CL=27.3\;*\;{\left(WT/70\right)}^{075}\;*\;\left(\frac{1}{1\;+\;{\left(\frac{TM_{50}}{PMA}\right)}^{Hill}}\right)$ *V*_1_ = 161 * (*WT*/70) *Q* = 26 * (*WT*/70)^0.75^ *V*_2_ = 7903 * (*WT*/70)

a *TM_50_ = 44.5 weeks; Hill = 2.56; F_inf_ = 0.73*.

b *TM_50_ = 33.7 weeks; Hill = 3.08*.

c *F_shunt_ = 1.24*.

d *TM_50_ = 41.9 weeks; Hill =7.04*.

*CL, clearance; CPB, cardiopulmonary bypass time; CMT, compartment; F_inf_, factor for children postoperative cardiac surgery; F_shunt_, factor for children with intracardiac right to left shunt; INR, internationalized normalized ratio; PMA, postmenstrual age; Q, intercompartmental clearance; TM_50_, age at which clearance reaches 50% of adult values; V, volume of distribution; V_1_, volume of distribution of the central compartment; V_2_, volume of distribution of the peripheral compartment; WT, weight*.

The population predicted concentrations were compared with the observed concentrations using prediction error (PE) as follows:


$$\begin{array}{l} PE=\frac{\left(C_{pred\;}-\;C_{obs\;}\right)}{C_{obs}}\;\times \;100 \end{array}$$

Where C_pred_ represents the concentration predicted by the model and C_obs_ represents the observed concentration in our study. The models' accuracy in predicting concentrations was determined using the median absolute PE (MDAPE), which represents the median of the absolute values of the PE, while the median PE (MDPE) was used to determine the bias of the predictions made by the models (18). Based on previously described acceptance criteria in the literature (19, 20), MDPE and MDAPE were considered acceptable at < |20| and 30%, respectively. Median errors (defined as C_pred_-C_obs)_ were also calculated. Moreover, the predictive performances of the models were visually evaluated using goodness of fit plots, depicting the population predicted vs. observed concentrations.

We then sought to determine if the discrepancies between population predicted and observed concentrations were better explained by differences in CL or V. To explore this, correction factors on CL and V were separately added to each of the six models, where the factors on CL and V were estimated, and the PK parameters and covariate effects were fixed [e.g., TVCL = THETA(1)^*^COVCL^*^THETA(2), where TVCL, THETA(1), and COVCL were fixed to the described parameters in the published models, respectively representing the typical value of CL in the described population, the parameter estimate for CL, and the covariate effect, and THETA(2) was estimated and represents the additional factor applied on CL]. The resulting population predicted concentrations were plotted against the observed concentrations for visual comparison.

The identified population PK models included inter-subject and residual variability, which were not taken into account using the prediction errors of the population predicted concentrations. To better compare observed concentrations with the range of expected concentrations based on the models, simulations were performed (1,000 replicates) for each of the six models. Graphical plots of the simulations were compared with plots of the actual data for visual inspection.

Analysis was performed using NONMEM (version 7.3.4) *via* the PDxPOP interface (version 5.2.2, ICON plc, Leopardstown, Dublin, Ireland). Output was summarized using STATA (version 14.2, StataCorp, College Station, Texas, USA), and figures were made using R (version 4.1.2, R Foundation for Statistical Computing, Vienna, Austria (www.r-project.org).

## Results

A total of 8 children contributed 30 samples between July 2018 and November 2019. Each subject contributed a median (range) of 3 samples (2–7) (Supplementary Figure 1). None of the included children were receiving renal replacement therapy. Most children (7/8) were supported by veno-arterial ECMO either for an acquired or congenital heart disease or a congenital diaphragmatic hernia (Table 2). Because of older age (15 years old compared to a median of 0.5 months) and different ECMO mode (veno-venous vs. veno-arterial), one subject was considered an outlier (subject 8), and analysis was performed with and without this subject.

**Table 2:** Demographics.

**Subject ID**	**Age (months)**	**Weight (kg)**	**Sex**	**ECMO type**	**Diagnosis**	**Infusion rate during PK sampling (mcg/kg/h)**	**Number PK samples**
1	13.2	7.9	M	VA	Acquired heart disease	0.5	3
2	6.1	7.1	F	VA	Post-Operative congenital heart disease	1	3
3	0.7	3.3	F	VA	Post-Operative congenital heart disease	0.7	4
4	0.5	3.8	F	VA	Post-Operative congenital heart disease	1	2
5	0.5	4.1	M	VA	Congenital diaphragmatic hernia	0.7–1	3
6	0.3	2.8	M	VA	Post-Operative congenital heart disease	0.6	2
7	0.8	4.5	F	VA	Congenital diaphragmatic hernia	0.6–1.1	6
8	181.5	35.9	F	VV	Respiratory failure	0.4–0.7	7

*ECMO, extracorporeal membrane oxygenation; PK, pharmacokinetic; VA, veno-arterial; VV, veno-venous*.

**Figure 1:**
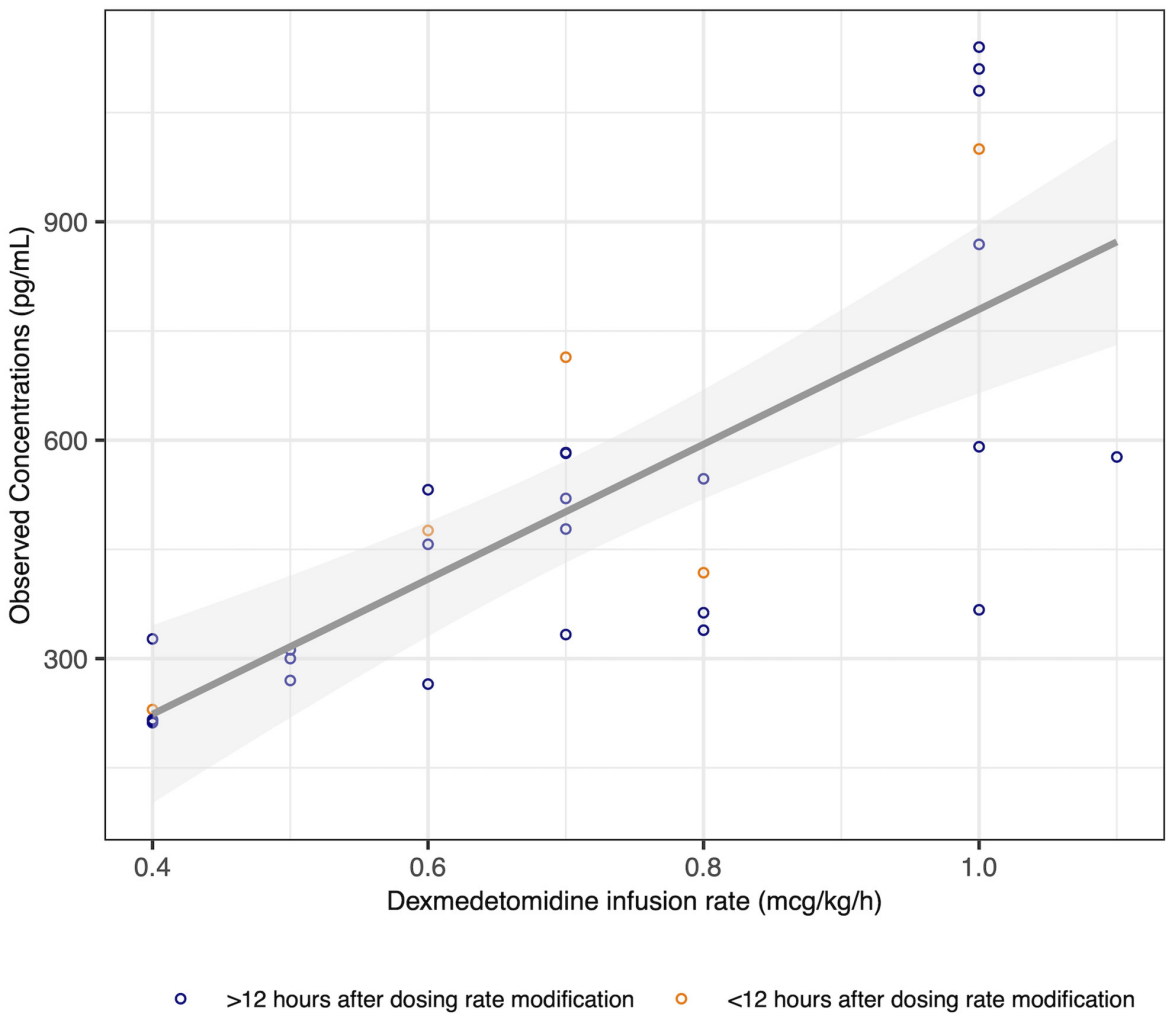
Dexmedetomidine concentrations vs. infusion rate.

In three subjects, a dexmedetomidine infusion was started before ECMO cannulation. Dexmedetomidine infusion rates ranged from 0.4 to 1.1 mcg/kg/h during PK sampling. In addition to the continuous infusion, 4 patients received dexmedetomidine boluses [total of 13 boluses received (0–8 per subject) with a median of 0.5 mcg/kg/dose (0.3–1.0)]. Samples were collected at a median of 14.9 days (1.6–40.7) following ECMO cannulation and 27.0 h (6.5–143.8) following dexmedetomidine initiation. Samples were collected at a median of 32.9 h (0.1–285.9) following a modification in the dexmedetomidine infusion rate, with 5 samples (17%) collected <12 h following the last modification to the infusion rate (Figure 1). Dexmedetomidine concentrations ranged from 212 to 1,140 pg/mL, and none of the samples were below the LLOQ (Figure 1, Supplementary Figure 1).

**Figure 2:**
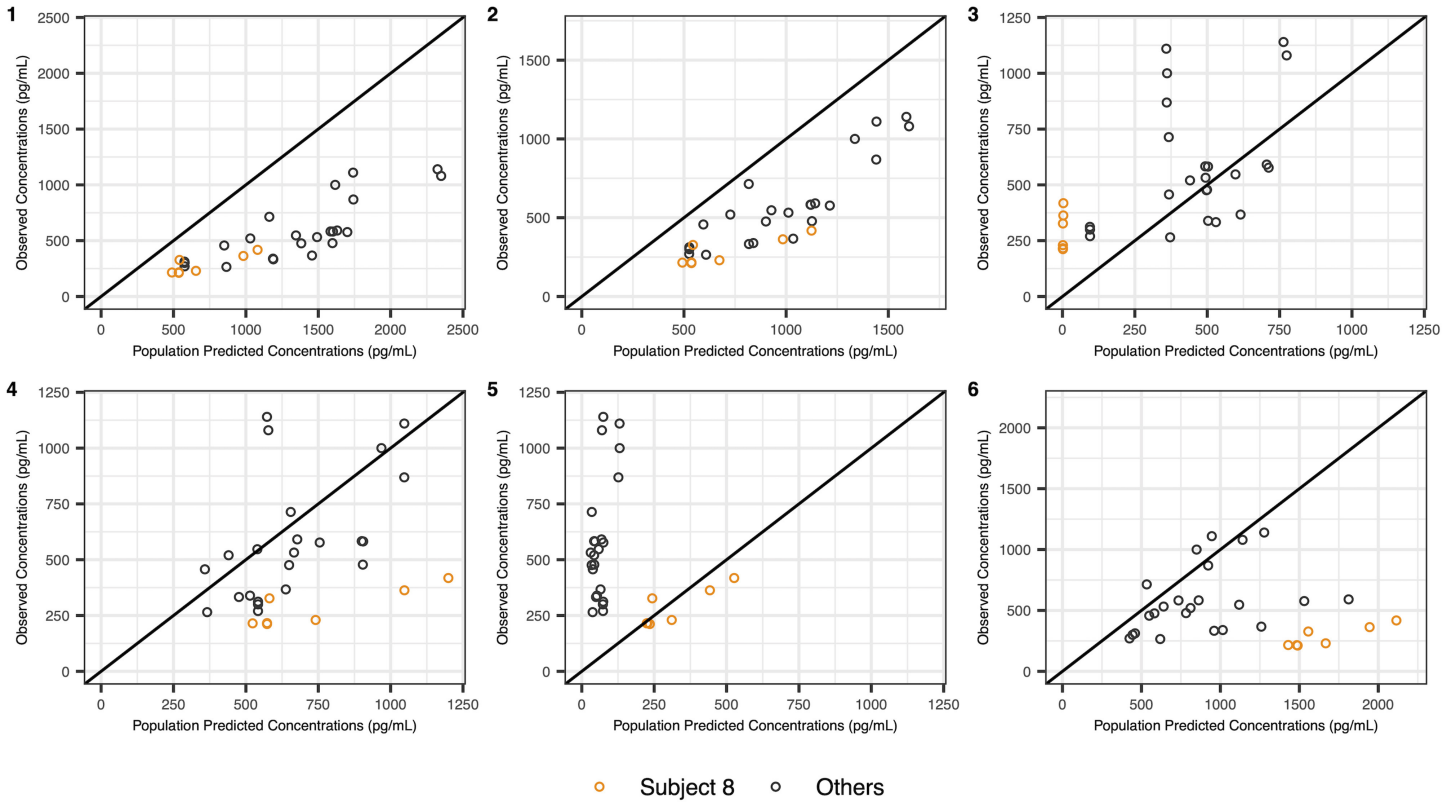
Observed concentrations vs. population predicted concentrations using previously published pharmacokinetics models in children. Observed concentrations vs. population predicted concentrations using population pharmacokinetics models developed by (1) Potts et al. (12), (2) van Dijkman et al. (13), (3) Greenberg et al. (14), (4) Su et al. (15), (5) Damian et al. (16), and (6) James et al. (17).

All tested models performed poorly in our full cohort, with MDPE ranging from −86 to 151% and MDAPE ranging from 51 to 151% (Supplementary Table 2). Models 1 and 2 consistently overpredicted the concentrations, while model 5 severely underpredicted the concentrations in all infants and neonates but was more accurate in our one adolescent subject (subject 8) (Figure 2). Model 3 performed best in neonates and infants on ECMO, with a MDPE of −15% and a MDAPE of 33% when excluding subject 8. Adding a factor on CL resulted in a better fit than the addition of a factor on V (Supplementary Figure 3). When looking at simulation plots (Supplementary Figures 4–9), most observed concentrations were within the range of the 90th prediction interval for models 3, 4, 5, and 6, while remaining below predicted concentrations for models 1 and 2.

## Discussion

This represents, to our knowledge, the first dexmedetomidine PK data in children on ECMO. Eight children aged 0.3 months to 15 years received a dexmedetomidine infusion ranging from 0.4 to 1.1 mcg/kg/min and achieved concentrations between 212 and 1,140 pg/mL. None of the previously described population PK models developed in children not supported by ECMO characterized the data with precision and acceptable bias. However, observed concentrations remained within the expected range predicted by four out of the six models evaluated.

The PK of different medications was found to be different in children on ECMO, with reduced CL and increased V frequently described (9, 21, 22). A decrease in CL leads to higher concentrations, while an increase in V translates into lower concentrations. Four of the evaluated models led to overprediction of the observed concentrations, which theoretically could have indicated increased V. However, adding a factor on V did not improve our goodness of fit plots, suggesting that increased V does not explain our findings. Most of our PK samples were collected at a steady state where concentrations depend mainly on infusion rate and clearance, which may explain why we could not identify a potentially increased V. However, an increased dexmedetomidine V during ECMO remains likely and may be caused by different factors. Children on ECMO often require blood products transfusions and intravenous fluids for circuit priming and maintaining hemodynamic stability. Adsorption from the ECMO circuit, specifically from the oxygenator, may also increase V and was described in previous dexmedetomidine *in vitro* studies (23, 24).

The addition of a correction factor on CL improved the goodness of fits plots, suggesting that an increase in CL explains our observed concentrations lower than the predicted ones. Although a decrease in CL is commonly encountered in critically ill children, increased CL has also been described with certain medications, notably antibiotics (25, 26). The main suggested mechanism for increased CL in critical care settings is augmented renal CL, which is common and often unrecognized in critically ill children (27). Given that dexmedetomidine is highly metabolized in the liver, with minimal unchanged excretion, changes in renal function are less likely to influence dexmedetomidine CL. However, increased CL on ECMO has been described with medications undergoing hepatic metabolism, including micafungin, clonidine, sildenafil, and midazolam (28–31). The impact of ECMO on metabolism pathways has not been well-characterized, but the high and continuous hepatic flow provided by the ECMO may conceivably increase the metabolism of medications with a high intrinsic CL. Moreover, dexmedetomidine is highly protein-bound, with a free fraction of only 6% (32). The free fraction of a medication is the only portion available for transport, metabolism, and excretion. Therefore, concomitant hypoalbuminemia, common in critically ill children (33), may also contribute to the augmented CL of dexmedetomidine by increasing its free fraction.

Interestingly, even though our observed concentrations were significantly different from the population predicted concentrations derived from the models, most of them were within the expected concentration range calculated using simulations based on the same models. This illustrates one of the pitfalls of population PK modeling: most models include such a high variability level that making accurate predictions is difficult. Moreover, when evaluating some of the models, the differences between predicted and measured concentrations exceeded the expected impact of ECMO, illustrating that models built in a specific population are often not applicable to different populations. This was the case with model 3 (14), which used an exponential equation to characterize the effect of postmenstrual age on CL. This model was robust when characterizing the PK in neonates and infants <7 months old. However, it assumes that CL keeps increasing exponentially with age and, therefore, severely underpredicted the concentrations obtained in our older subject. Conversely, model 5 (16), which does not include any “size” parameter on CL, characterized dexmedetomidine disposition relatively well in our one adolescent while severely underpredicting concentrations in our younger subjects. Those models' structures render population PK models challenging to apply in clinical settings, especially in pediatric critical care where a wide range of ages, weights, pathologies, and extracorporeal devices is present.

Dexmedetomidine concentrations between 212 and 1,140 pg/mL were achieved in our cohort. This study was not designed to correlate concentrations with achieved sedation. However, clinicians could titrate dexmedetomidine infusion rates as needed, suggesting that this concentration range yielded satisfactory sedation as judged by the bedside clinicians. Concentrations above 700 pg/mL were associated with sedation in healthy adult men (34). This threshold was achieved in three children with infusion rates between 0.7 and 1 mcg/kg/h, although equivalent doses yielded lower concentrations in three other children. As expected, observed concentrations increased with dexmedetomidine infusion rates, as shown in Figure 1. Considering that the maximum dosing rate in our study was 1.1 mcg/kg/h, our results suggest that the proposed threshold of concentration >700 pg/mL is attainable in children on ECMO while staying within the dosing range of 0.1 and 2.5 mcg/kg/h reported in critically ill children (35). However, the exact dexmedetomidine concentrations leading to adequate sedation in an acute care setting remain to be determined, especially in children on ECMO, considering the more invasive equipment and severity of the disease contributing to greater discomfort.

Our study is exploratory and has significant limitations. Our limited sample size and sampling precluded us from determining the PK of dexmedetomidine in children on ECMO and only allowed a comparison with available data in children without ECMO. While we were able to show that concentrations corresponding to adequate sedation based on previous studies were reached using standard infusion rates, the achieved sedation status was not assessed in the current study. Indeed, this study was not designed to establish the pharmacodynamics of dexmedetomidine, and more studies are needed to better determine the concentrations needed to achieve adequate sedation in critically ill children. Finally, the patients included did not receive standardized dexmedetomidine infusion rates.

A detailed characterization of the distribution and the elimination of dexmedetomidine in children supported with ECMO is needed. However, such studies are difficult to perform. Assuming an arbitrary coefficient of variation of 60% on clearance, at least 16 patients per age group would be needed to develop a population PK model with 80% power (36). Using this rough estimate, between 50 and 100 children would have to be included to describe dexmedetomidine PK over the whole pediatric age spectrum, depending on how many age groups are determined. The required sample size may even be higher depending on the planned sampling scheme and the model's complexity. Considering the inherent difficulties in recruiting children for PK studies on ECMO, added to the relatively infrequent use of dexmedetomidine during ECMO, performing such an ambitious study appears quite challenging and perhaps unrealistic. However, studies in specific age groups such as neonates, which comprise a significant portion of the pediatric ECMO population, appear possible, notably through multicenter collaboration.

## Conclusion

This study includes preliminary data on dexmedetomidine PK in children on ECMO. The population PK models developed in children not supported by ECMO poorly predicted dexmedetomidine PK in our population. Most models overpredicted the concentrations, which may represent increased CL. Larger studies are needed to characterize the disposition of dexmedetomidine on ECMO. Specificities of population PK models may restrict their use to a limited population. Future studies better reflecting the whole spectrum of the pediatric critical care population would be helpful to facilitate the application of PK models into clinical care, although challenging to perform.

## Data Availability Statement

The raw data supporting the conclusions of this article will be made available by the authors, without undue reservation.

## Ethics Statement

The studies involving human participants were reviewed and approved by Children's Hospital of Philadelphia Review Board. Written informed consent to participate in this study was provided by the participants' legal guardian/next of kin. Written informed consent was obtained from the minor(s)' legal guardian/next of kin for the publication of any potentially identifiable images or data included in this article.

## Author Contributions

CT and AZ designed the study, and drafted, reviewed, and revised the manuscript. CT collected and analyzed the data. All authors approved the final manuscript as submitted and agree to be accountable for all aspects of the work.

## Conflict of Interest

The authors declare that the research was conducted in the absence of any commercial or financial relationships that could be construed as a potential conflict of interest.

## Publisher's Note

All claims expressed in this article are solely those of the authors and do not necessarily represent those of their affiliated organizations, or those of the publisher, the editors and the reviewers. Any product that may be evaluated in this article, or claim that may be made by its manufacturer, is not guaranteed or endorsed by the publisher.
