# Supplementary material for: Dexmedetomidine in Children on Extracorporeal Membrane Oxygenation: Pharmacokinetic Data Exploration Using Previously Published Models
Source: Front Pediatr. 2022 Jun 27;10:924829. doi: 10.3389/fped.2022.924829 (PMC9271626; doi:10.3389/fped.2022.924829)
Supplement: Supplementary file 1 [file Data_Sheet_1.PDF]

Supplemental Figure 1. Dexmedetomidine concentrations vs. infusion rate by subject ID

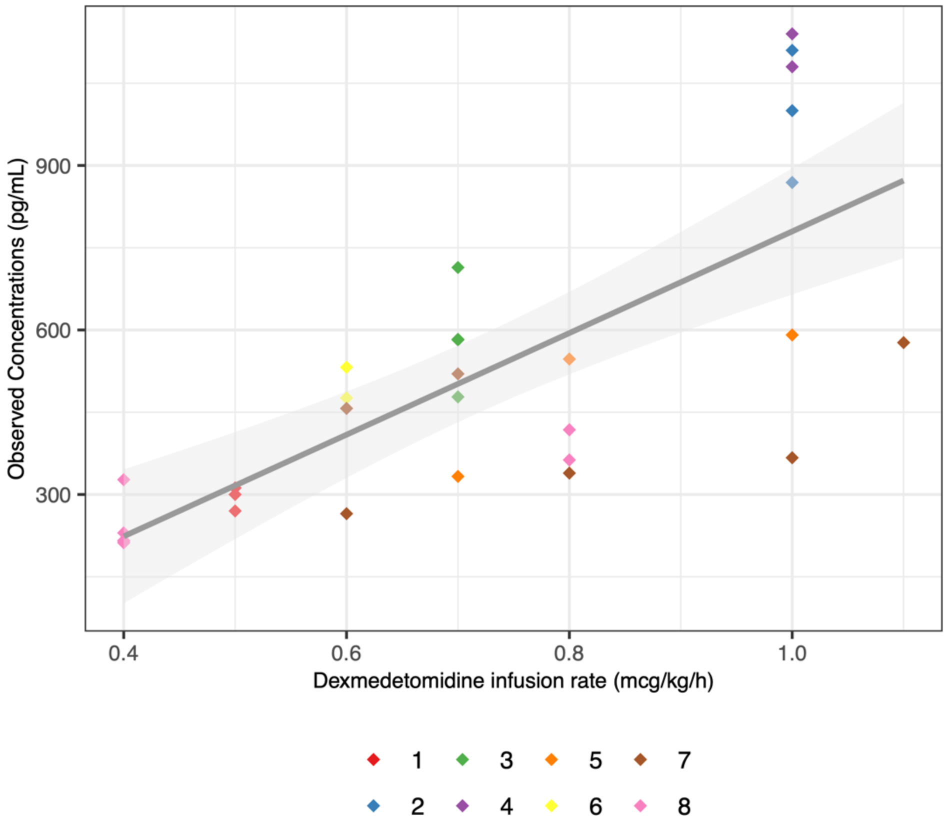

Supplemental Table 2. Prediction errors of the evaluated models

Analysis	Models	MDPE[1] (%)	MDAPE[1] (%)	ME[1] (pg/mL)
**Full cohort**	1 (Potts et al.)	151 (98, 185)	151 (98, 184)	647 (393, 1002)
	2 (van Dijkman et al.)	92 (66, 146)	92 (66, 146)	434 (277, 538)
	3 (Greenberg et al.)	-31 (-70, 5)	54 (19, 70)	-190 (-325, 22)
	4 (Su et al.)	47 (-1, 89)	51 (22, 89)	178 (-7, 317)
	5 (Damian et al.)	-86 (-92, -73)	86 (73, 92)	-427 (-536, -197)
	6 (James et al.)	60 (20, 243)	60 (22, 243)	298 (108, 1213)
**Excluding subject 8**	1 (Potts et al.)	146 (92, 195)	146 (92, 195)	857 (511, 1040)
	2 (van Dijkman et al.)	89 (40, 110)	89 (40, 100)	448 (255, 539)
	3 (Greenberg et al.)	-15 (-59, 19)	33 (15, 64)	-79 (-306, 107)
	4 (Su et al.)	31 (-6, 56)	38 (15, 56)	143 (-59, 242)
	5 (Damian et al.)	-88 (-93, -85)	88 (85, 93)	-489 (-680, -287)
	6 (James et al.)	48 (12, 134)	48 (20, 134)	153 (92, 572)

[1]Median and interquartile ranges

MDAPE: median absolute prediction error, MDPE: median prediction error; ME: median error

Supplemental Figure 3. Observed concentrations versus population predicted concentrations

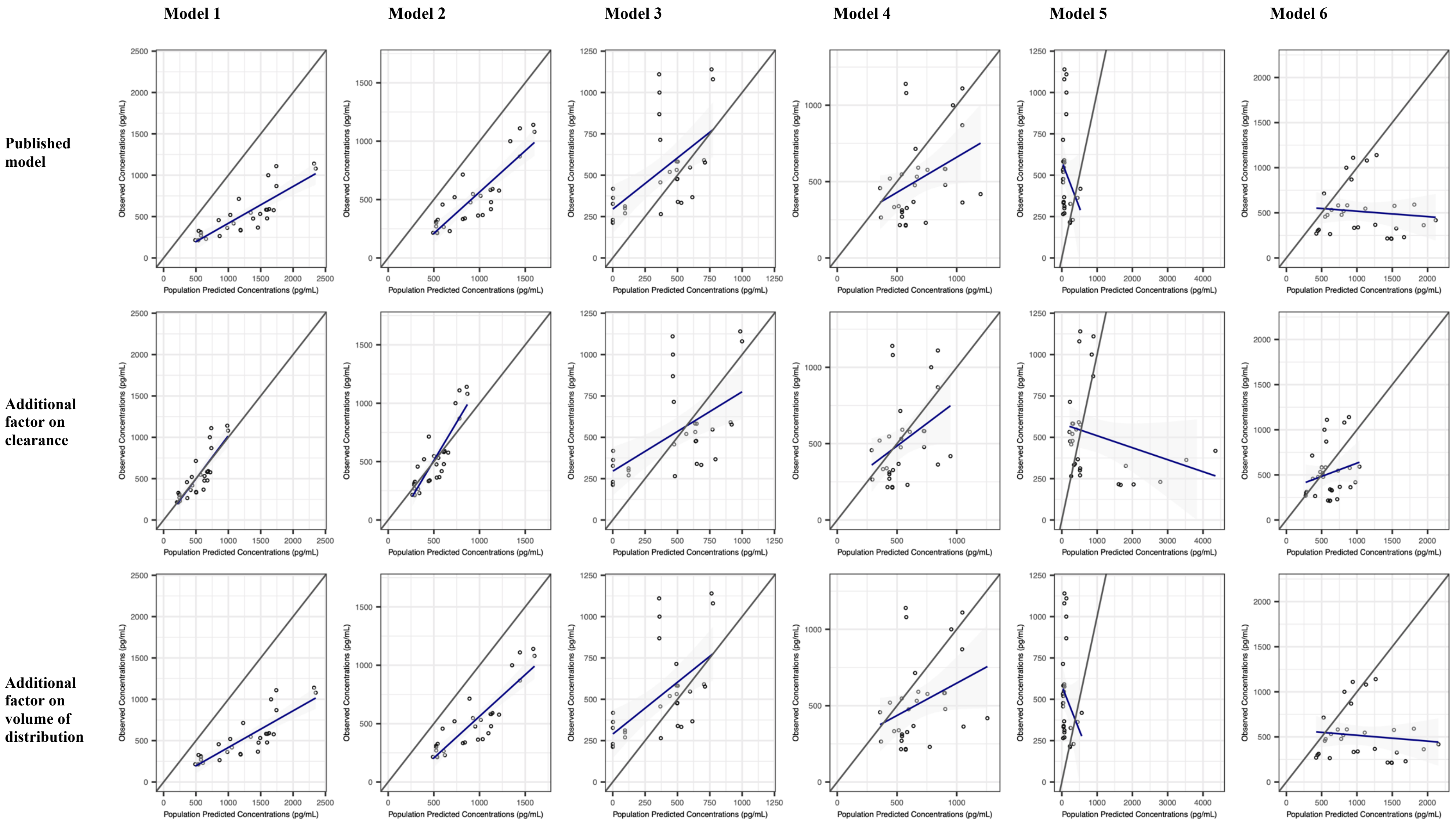

Observed vs. population predicted concentrations using the published model (first row), and adding a factor on clearance (second row) and on volume of distribution (third row).

Supplemental Figure 4. Simulations using Model 1 (Potts et al.)

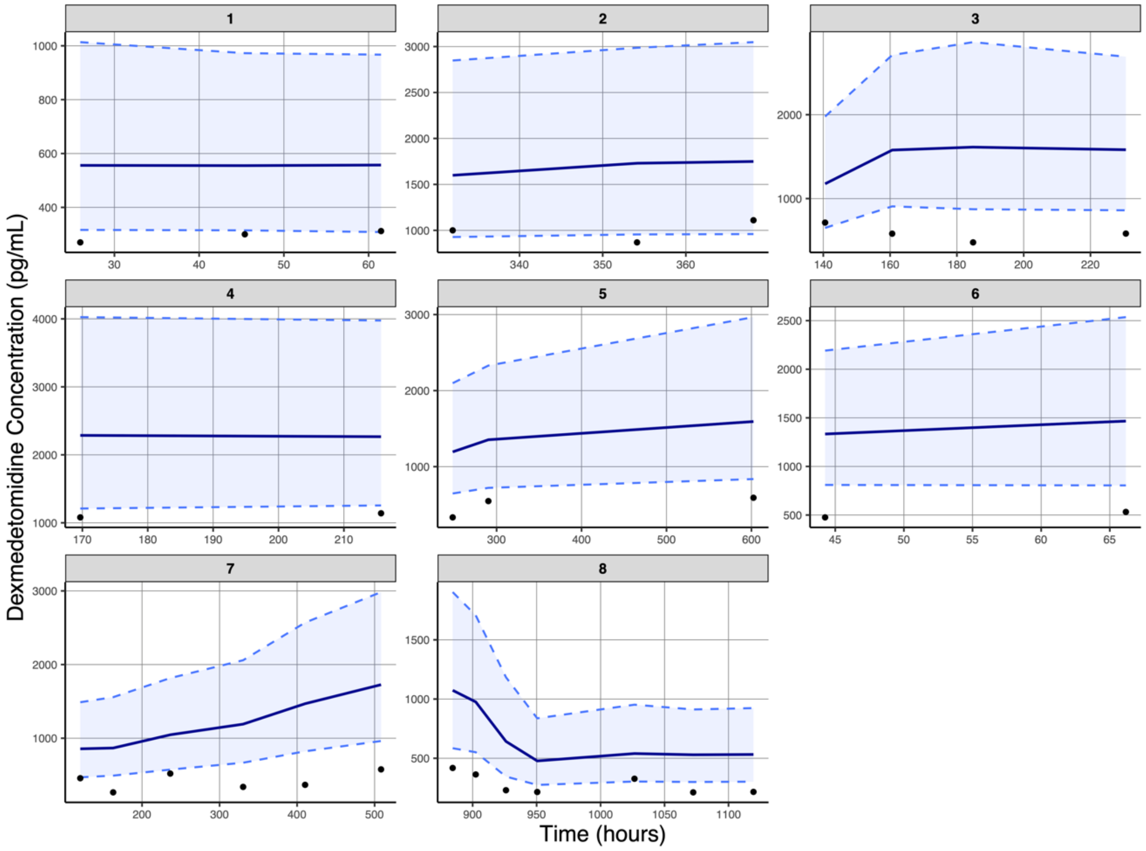

The dots represent the observed concentrations. The navy line represents the median, and the dotted lines represent the 5th and 95th percentiles of the simulated concentrations.

Supplemental Figure 5. Simulations using Model 2 (van Dijkman et al.)

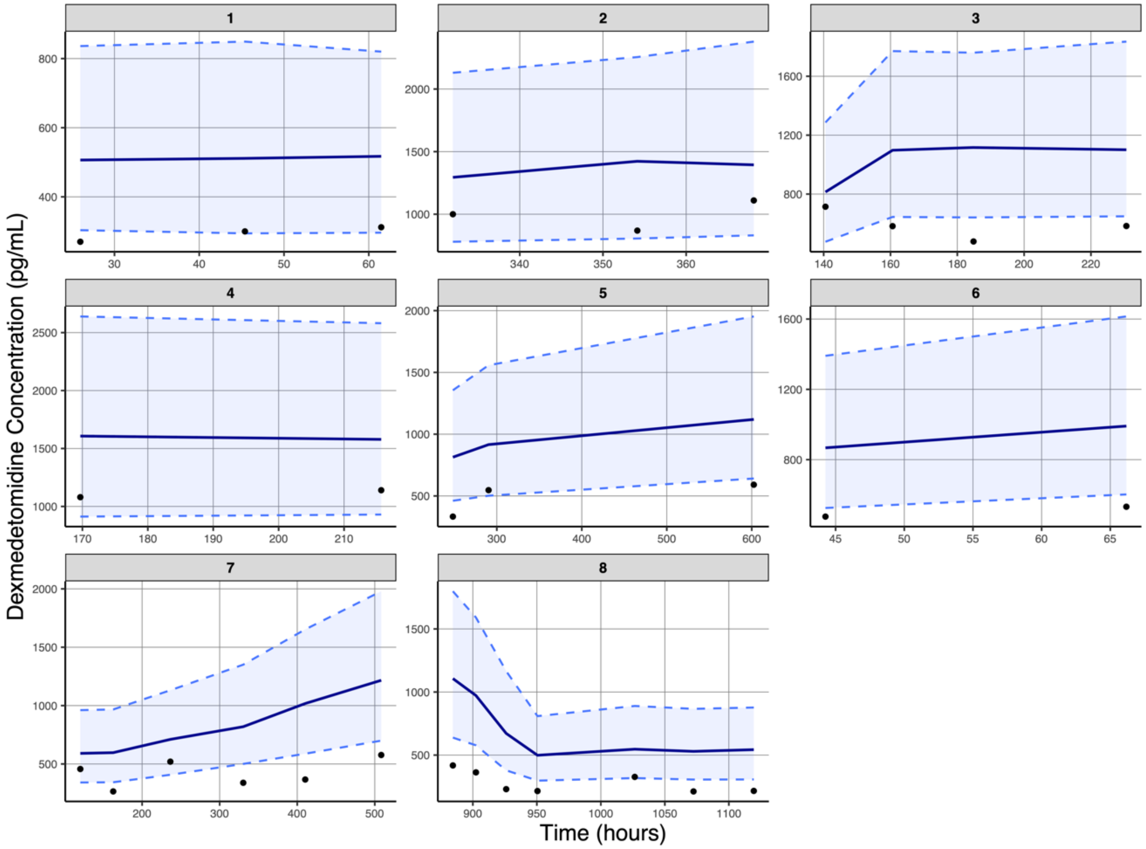

The dots represent the observed concentrations. The navy line represents the median, and the dotted lines represent the 5th and 95th percentiles of the simulated concentrations.

Supplemental Figure 6. Simulations using Model 3 (Greenberg et al.)

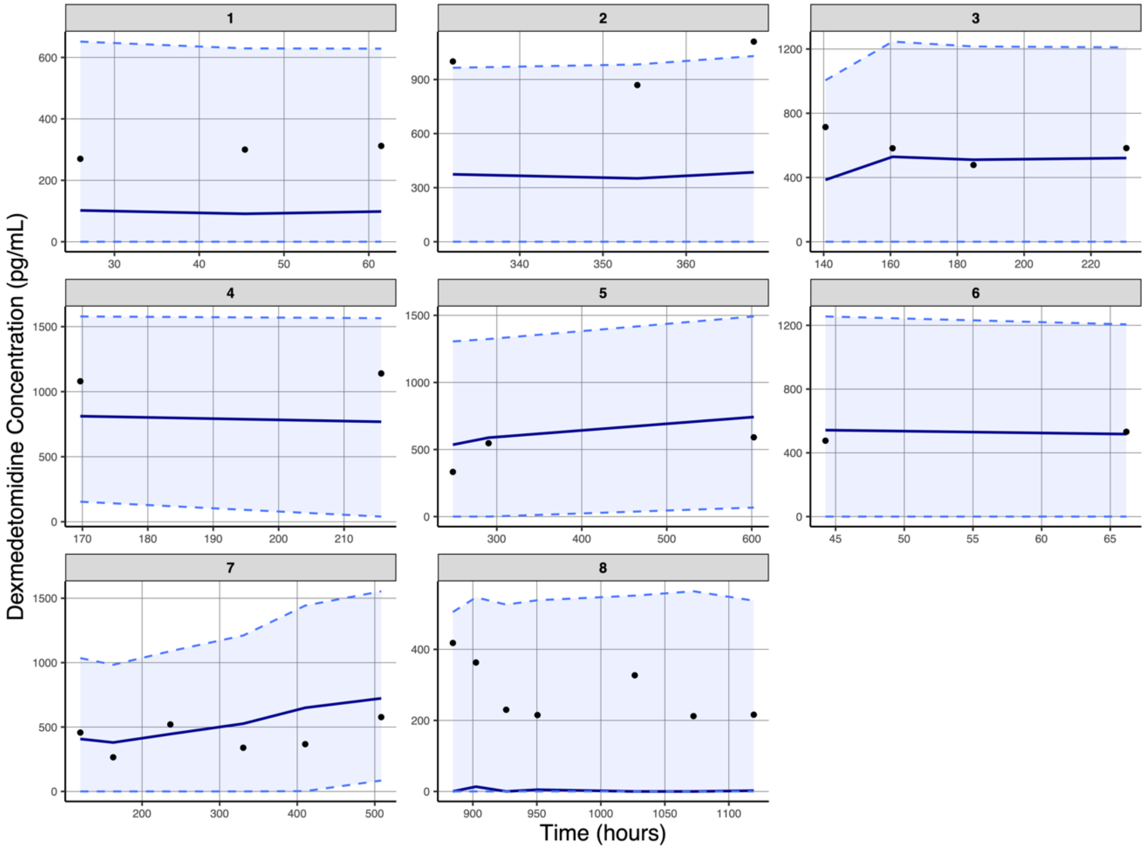

The dots represent the observed concentrations. The navy line represents the median, and the dotted lines represent the 5th and 95th percentiles of the simulated concentrations.

Supplemental Figure 7. Simulations using Model 4 (Su et al.)

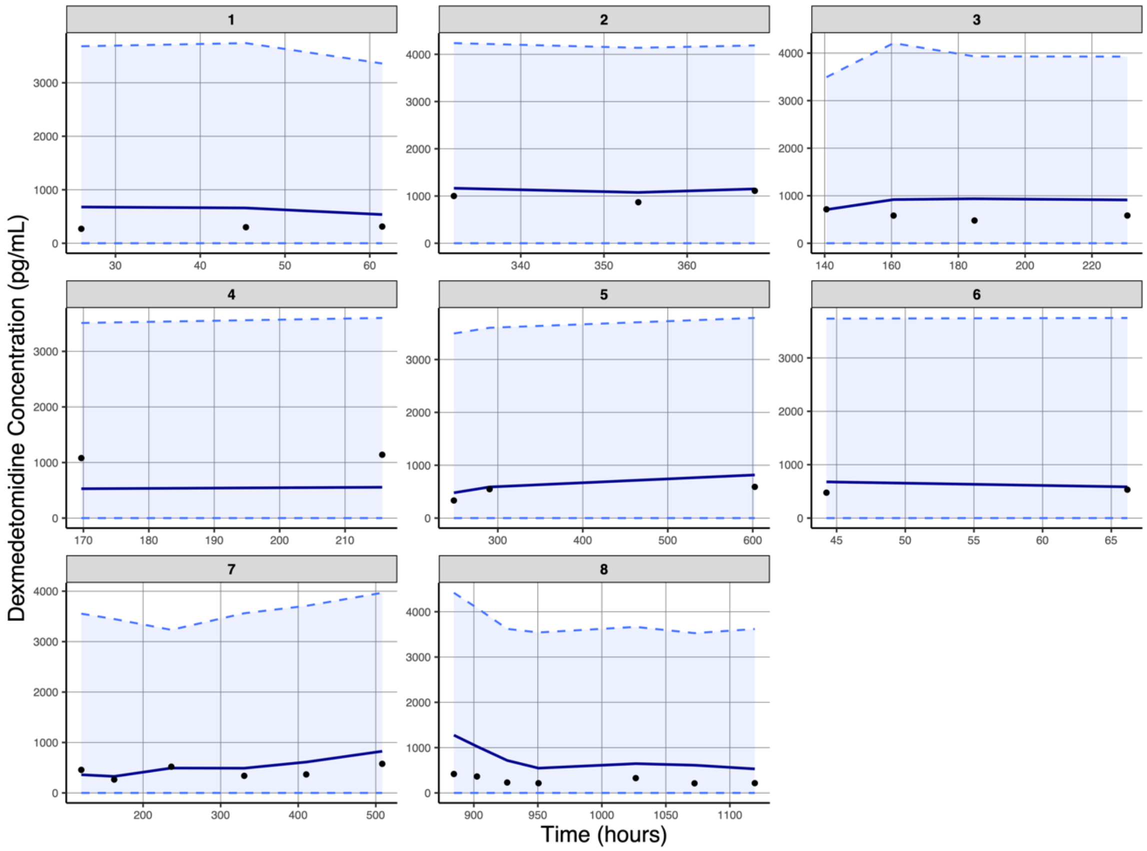

The dots represent the observed concentrations. The navy line represents the median, and the dotted lines represent the 5th and 95th percentiles of the simulated concentrations.

Supplemental Figure 8. Simulations using Model 5 (Damian et al.)

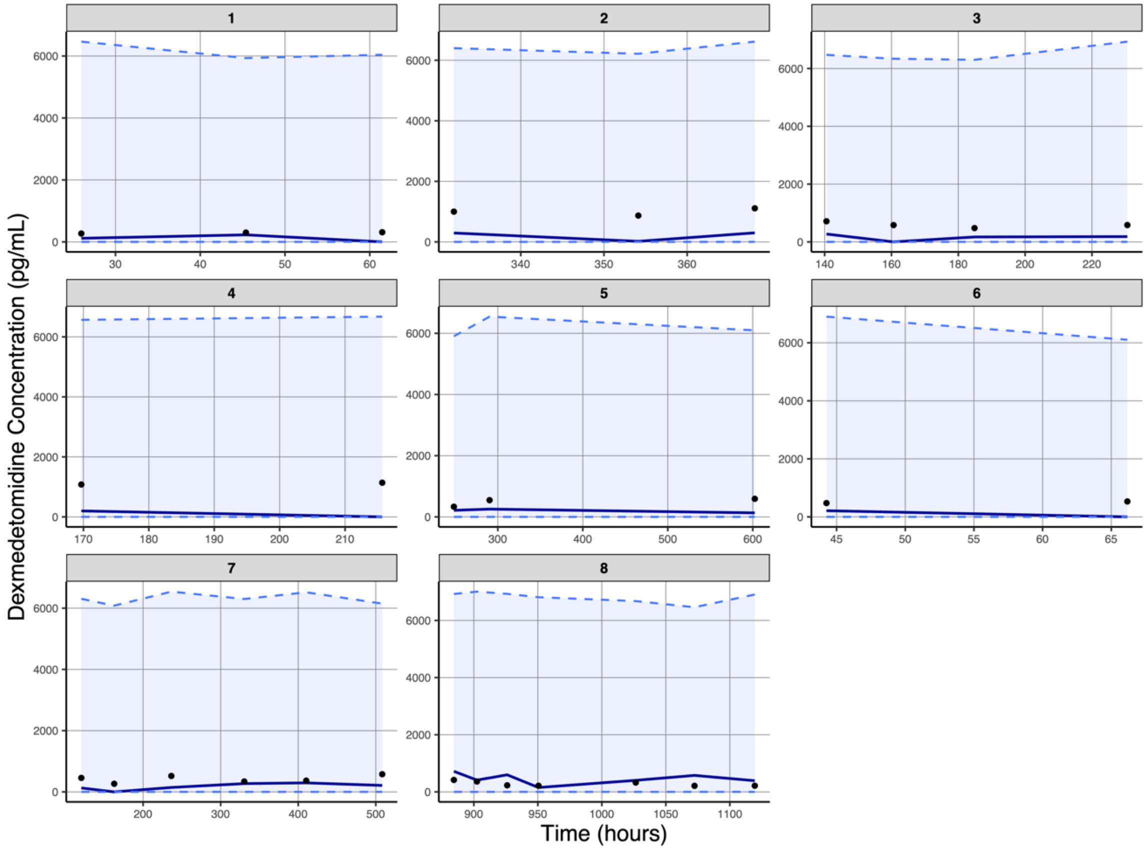

The dots represent the observed concentrations. The navy line represents the median, and the dotted lines represent the 5th and 95th percentiles of the simulated concentrations.

Supplemental Figure 9. Simulations using Model 6 (James et al.)

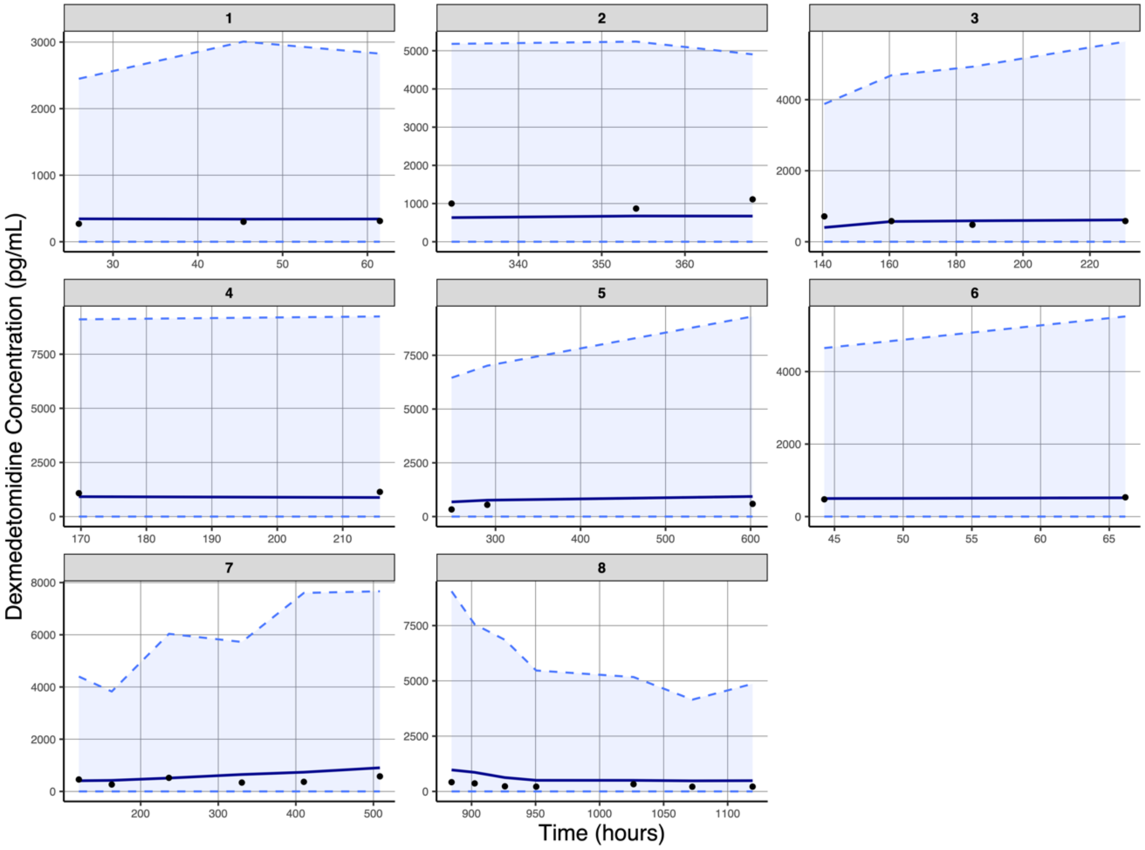

The dots represent the observed concentrations. The navy line represents the median, and the dotted lines represent the 5_{th} and 95_{th} percentiles of the simulated concentrations.
